# The PD-L1 metabolic interactome intersects with choline metabolism and inflammation

**DOI:** 10.1186/s40170-021-00245-w

**Published:** 2021-02-19

**Authors:** Jesus Pacheco-Torres, Marie-France Penet, Yelena Mironchik, Balaji Krishnamachary, Zaver M. Bhujwalla

**Affiliations:** 1 Division of Cancer Imaging Research, The Russell H. Morgan Department of Radiology and Radiological Science, The Johns Hopkins University School of Medicine, 720 Rutland Avenue, Rm 208C Traylor Building, Baltimore, MD 21205 USA; 2 Sidney Kimmel Comprehensive Cancer Center, The Johns Hopkins University School of Medicine, Baltimore, MD 21205 USA; 3 Department of Radiation Oncology and Molecular Radiation Sciences, The Johns Hopkins University School of Medicine, Baltimore, MD 21205 USA

**Keywords:** Immune checkpoints, PD-L1, Choline kinase alpha, COX-2, Breast cancer

## Abstract

**Background:**

Harnessing the power of the immune system by using immune checkpoint inhibitors has resulted in some of the most exciting advances in cancer treatment. The full potential of this approach has, however, not been fully realized for treating many cancers such as pancreatic and breast cancer. Cancer metabolism influences many aspects of cancer progression including immune surveillance. An expanded understanding of how cancer metabolism can directly impact immune checkpoints may allow further optimization of immunotherapy. We therefore investigated, for the first time, the relationship between the overexpression of choline kinase-α (Chk-α), an enzyme observed in most cancers, and the expression of the immune checkpoint PD-L1.

**Methods:**

We used small interfering RNA to downregulate Chk-α, PD-L1, or both in two triple-negative human breast cancer cell lines (MDA-MB-231 and SUM-149) and two human pancreatic ductal adenocarcinoma cell lines (Pa09C and Pa20C). The effects of the downregulation were studied at the genomic, proteomic, and metabolomic levels. The findings were compared with the results obtained by the analysis of public data from The Cancer Genome Atlas Program.

**Results:**

We identified an inverse dependence between Chk-α and PD-L1 at the genomic, proteomic, and metabolomic levels. We also found that prostaglandin-endoperoxide synthase 2 (COX-2) and transforming growth factor beta (TGF-β) play an important role in this relationship. We independently confirmed this relationship in human cancers by analyzing data from The Cancer Genome Atlas Program.

**Conclusions:**

Our data identified previously unknown roles of PD-L1 in cancer cell metabolic reprogramming, and revealed the immunosuppressive increased PD-L1 effect of Chk-α downregulation. These data suggest that PD-L1 regulation of metabolism may be mediated through Chk-α, COX-2, and TGF-β. The observations provide new insights that can be applied to the rational design of combinatorial therapies targeting immune checkpoints and cancer metabolism.

**Supplementary Information:**

The online version contains supplementary material available at 10.1186/s40170-021-00245-w.

## Background

The cancer immunotherapy field has been revitalized with the discovery of immune checkpoints such as programmed cell death protein-1 (PD-1), and its ligand (PD-L1, *CD274*), that act as natural regulators of the immune system [1]. Along with the expanded interest in blocking immune checkpoints for cancer immunotherapy, recent studies have also identified the important role of metabolism in immune suppression and the tumor immune microenvironment (TIME) [2, 3]. Cancer cells can create an immune-suppressive microenvironment through metabolic reprogramming, by changing the metabolic profile of the tumor microenvironment (TME) and exerting high metabolic stress on tumor-infiltrating immune cells that lead to their functional inactivation [4–7]. High glycolysis and lactate production [8, 9], glutamine deprivation [10], and changes in the redox state [11] have been shown to influence the TIME. PD-L1 levels are, furthermore, directly modulated in cancer cells by lactate [12], arginine [13], or glutamate [14], and in immune cells by arginine [13] and glutamine [15]. As a result, metabolic inhibitors of the glutamate, glutamine, and arginine pathways are being evaluated in clinical trials in combination with immune checkpoint inhibitors with promising outcomes [6].

Most cancers exhibit an aberrant choline metabolism that is characterized by increased phosphocholine (PC) and total choline-containing compounds [16, 17]. These changes have been attributed, in large part, to the overexpression and increased activity of choline kinase (Chk)-α, encoded by *CHKA*, in malignant cells [18, 19]. Chk-α catalyzes the phosphorylation of choline (Cho) to PC, a major component of phosphatidylcholine (PtdCho) cycle. PtdCho is the most abundant phospholipid of the eukaryotic cell membrane and an important source of signaling molecules [20]. The prognostic and oncogenic roles of Chk-α have been described in studies with different tumor types [21–23], relating Chk-α overexpression to tumor progression, metastasis, and the activation of oncogenic signaling pathways [24]. As a result, Chk-α inhibitors are being evaluated as therapeutic agents [25, 26], with at least one that has recently completed a phase I clinical trial [27]. Despite being one of the most consistently altered metabolic pathway in cancers, the relationship between choline metabolism and immune checkpoints has, to date, not been investigated. New insights into the interaction between choline metabolism and immune checkpoints may lead to improved treatment outcomes.

Here, for the first time, we investigated the relationship between choline metabolism and the immune checkpoint PD-L1, in human triple-negative breast cancer (TNBC) and pancreatic ductal adenocarcinoma (PDAC) cells. We focused on TNBC and PDAC because the outcomes of immune checkpoint inhibitor treatments in these cancers have yet to match the successes observed in melanoma and lung cancer [28, 29]. PDAC, especially, has poor survival rates [30]. Chk-α and PD-L1 were downregulated using Chk-α- and PD-L1-specific small interfering RNA (siRNA). The functional impact of Chk-α and PD-L1 downregulation on the PD-L1 metabolic interactome was characterized by performing high-resolution ^1^H magnetic resonance spectroscopy (MRS) of cell extracts to quantify changes in metabolic patterns. We found an inverse dependence between Chk-α and PD-L1 levels in three of the four cancer cell lines investigated that was mediated, in part, by prostaglandin-endoperoxide synthase 2 (COX-2) and transforming growth factor beta (TGF-β). The multiple metabolic changes observed when Chk-α and PD-L1 were downregulated individually were largely attenuated when both were downregulated together, further supporting the important role of Chk-α in the PD-L1 metabolic interactome and vice versa. These results were independently verified in human cancers by analysis of data from The Cancer Genome Atlas Program (TCGA) that confirmed a significant negative correlation between Chk-α and PD-L1 expression.

## Methods

### Cell lines

Triple-negative MDA-MB-231 human breast cancer cells (ATCC Cat# HTB-26, RRID: CVCL_0062, female) were obtained from ATCC (Manassas, VA, USA). MDA-MB-231 cells with COX-2 silenced (shCOX2-MDA-MB-231) were created through the use of a short hairpin RNA-coding plasmid constructed and placed under the control of the U6 promoter as previously described [31]. Triple-negative SUM-149 human breast cancer cells (CVCL_3422, RRID: CVCL_3422, female) were obtained from Asterand (Asterand Inc., Detroit, MI, USA). Pa09C (Panc215, RRID: CVCL_E286, female) and Pa20C (Panc198/RRID: CVCL_E285, male) human pancreatic cancer cells obtained from primary PDAC were kindly provided by Dr. Anirban Maitra [32]. All cell lines were authenticated within the past 6 months at the Johns Hopkins Genetic Resource Core Facility that follows ASN-0002-2011, Authentication of Human Cell Lines: Standardization of STR Profiling guidelines. The STR profiles of MDA-MB-231 and shCOX2-MDA-MB-231 cells were verified using the ATCC database, and the STR profile of SUM-149 cells was verified using the ExPASY Bioinformatics Resource Portal in the Cellosaurus database [33]. STR profiling of the pancreatic cancer cell lines confirmed the absence of cross-contamination. All the cell lines used in this study tested negative for mycoplasma within the past 6 months.

### Cell culture

Cells were cultured under standard cell culture incubator conditions at 37 °C in a humidified atmosphere containing 5% CO_2_ and were used from passages 3–7. MDA-MB-231 cells were maintained in RPMI 1640 medium (Sigma-Aldrich, St. Louis, MO, USA) supplemented with 10% fetal bovine serum (FBS, Sigma-Aldrich,). shCOX2-MDA-MB-231 cells were maintained in RPMI 1640 medium supplemented with 10% FBS and G418 sulfate (Corning, Corning, NY, USA) at a concentration of 400 μg/mL. SUM-149 cells were maintained in DMEM/Ham’s F12 50/50 medium (Sigma-Aldrich) with 5% calf serum, insulin (5 μg/ml), and hydrocortisone (1 μg/mL). Pa09C cells were cultured in RPMI-1640 with 20% FBS, 12.5 mmol/L glucose, and 2 mmol/L glutamine. Pa20C cells were cultured in DMEM (Sigma-Aldrich) with 10% FBS, 25 mmol/L glucose, and 4 mmol/L glutamine.

### RNA interference experiments

All siRNA were purchased from Dharmacon (Lafayette, CO, USA). Untreated cells and cells treated with nontargeted scrambled siRNA (Dharmacon, Catalog Item D-001810-10-20) or luciferase siRNA (Dharmacon, Catalog Item P-002099-01-50) were used as controls. Isoform-specific siRNAs were custom designed using Thermo Scientific siRNA Design Center (Thermo Scientific, Rockford, IL, USA). siRNA specific sequences were 5′-CAUGCUGUUCCAGUGCUCC-3′ for Chk-α, 5′-GAGGAAGACCUGAAGGUUCAGCAUA-3′ for PD-L1 #1, and 5′-CCUACUGGCAUUUGCUGAACGCAUU-3′ for PD-L1 #2.

Cells at 40 to 50% confluency were transfected with 100 nM of scrambled or luciferase siRNA, and with 50 nM or 100 nM of Chk-α- or PD-L1-specific siRNA for individual treatments. For combination siRNA treatments, 50 nM of each specific siRNA was used. Cells were treated with siRNA for 48 h because this incubation period resulted in the most effective downregulation of the target genes. D-FECT 4 (Dharmacon, Catalog Item T-2004-03) was used as the transfection agent for MDA-MB-231, Pa09C and Pa20C cells, and Lipofectamine 2000 (Thermo Fisher, Waltham, MA, USA, Catalog Item 11668019) for SUM-149 cells. All transfections were carried out based on established protocols [34].

### RNA isolation, cDNA synthesis, and RT-PCR

Approximately 0.4 × 10^6^ cells were incubated with different siRNA as previously described [34]. Total RNA was isolated from cells using the QIAshredder and RNeasy Mini kit (Qiagen, Valencia, CA, USA) as per the manufacturer’s protocol. cDNA was prepared using the iScript cDNA synthesis kit (Bio-Rad, Hercules, CA, USA). Real-time PCR of cDNA samples was performed using IQ SYBR Green supermix and gene-specific primers in the iCycler real-time PCR detection system (Bio-Rad). All primers were designed using either the Beacon designer software 7.8 (Premier Biosoft, Palo Alto, CA, USA) or the publicly available Primer3plus software. The expression of target RNA relative to the housekeeping gene hypoxanthine phosphoribosyltransferase 1 (HPRT1) was calculated based on the threshold cycle (C_t_) as *R* = 2-^Δ(ΔCt)^, where ΔC_t_ = C_t_ of target gene - C_t_ of HPRT1 and Δ(ΔC_t_) = ΔC_t_ siRNA treated cells - ΔC_t_ untreated cells.

### Protein isolation and immunoblots

Approximately 10^6^ cells were incubated with different siRNA for 24 h, 48 h, and 72 h. Total protein was extracted using a 1× cracking buffer [100 mmol/L Tris (pH 6.7), 2% glycerol] containing a protease inhibitor (Sigma) at 1:200 dilution. Protein concentration was estimated using the Bradford Bio-Rad protein assay Kit (Bio-Rad). Approximately 100 μg of total protein was used in each experiment. Expression levels of Chk-α, PD-L1, and COX-2 were determined by immunoblotting using a custom-made polyclonal antibody against Chk-α at 1:200 dilution, a rabbit polyclonal against human PD-L1 at 1:1000 dilution (GeneTex, Irvine, CA, Cat# GTX104763, RRID: AB_1240586), and a goat anti-COX-2 antibody at 1:500 dilution (Cayman Chemical, Ann Arbor, Michigan, Cat# 100034, RRID: AB_10078977). Monoclonal anti-GAPDH antibody (1:50,000 dilution, Sigma-Aldrich, Cat# G8795, RRID: AB_1078991) was used as a loading control. Proteins were visualized with HRP (horseradish peroxidase)-conjugated secondary antibodies using the SuperSignal West Pico Chemiluminescent substrate kit (Thermo Scientific).

### Prostaglandin E2 concentration

Prostaglandin E_2_ (PGE_2_) concentrations were measured as previously described [35] using the supernatant of cells under the different treatment conditions. PGE_2_ enzyme immunoassay (EIA) Kit-Monoclonal was used as described by the manufacturer (Cayman Chemical, Ann Arbor, MI).

### Flow cytometry analysis of PD-L1

Approximately 10^6^ cells were transfected with siRNA for 48 h. The following antibodies were used for flow cytometry: APC mouse anti-human PD-L1 (CD274; clone MIH1 from BD Pharmingen, San Diego, CA, USA) and APC mouse IgG1 isotype (Clone MOPC-21, BD Pharmingen). Briefly, cells were washed and harvested using PBS EDTA 5 mM buffer. Approximately 0.5 × 10^6^ cells were suspended in PBA buffer (PBS containing 0.5% bovine serum albumin and 0.02% sodium azide). Cells were incubated with either PD-L1 or IgG1 antibody in the dark at 4 °C for 1 h and washed several times in PBA. Flow analyses were performed using a FACS Calibur system (Becton Dickinson Immunocytometry Systems, San Jose, CA, USA). IgG1 controls were analyzed to delineate the negative population and to designate the quadrants. The percentage of positive events and the mean fluorescent intensity was measured using the Cell Quest software (Becton Dickinson Immunocytometry Systems).

### Dual-phase extraction and high-resolution ^1^H MRS

Approximately 2 × 10^7^ cells were incubated with different siRNA for 48 h. Water-soluble and lipid fractions were extracted from the cells using a dual-phase extraction method [36]. Briefly, pelleted cells were washed with ice-cold saline, then mixed with 4 mL of ice-cold methanol and vigorously vortexed. After keeping samples on ice for 15 min, 4 mL of chloroform was added, vortexed vigorously, and kept on ice for an additional 10 min. Finally, 4 mL of water was added, and the samples were vortexed again. All procedures were performed on ice, and samples were stored at 4 °C overnight for phase separation and then centrifuged at 15,000×*g* at 4 °C for 30 min. The aqueous phase containing water-soluble metabolites was collected [37]. Methanol in the aqueous phase was first evaporated under nitrogen gas, and any water remaining in the aqueous phase was lyophilized. Dried aqueous phase extracts were re-suspended in 0.6-mL deuterated water (D_2_O) for MRS analysis. 3-(Trimethylsilyl) propionic 2,2,3,3-d4 acid sodium salt (TSP) dissolved in D_2_O was used as an internal standard. Lipid phase extracts were dried under nitrogen gas stream and re-suspended in 0.6-mL deuterated chloroform and methanol in a 2:1 ratio containing tetramethylsilane (TMS) 0.05% v/v.

High-resolution ^1^H MR spectra were recorded on a Bruker Biospin Avance-III 750 MHz NMR (Bruker Biospin Billerica, MA, USA) spectrometer operating at a proton frequency of 750.21 MHz using a 5-mm broad band inverse (BBI) probe equipped with z-gradient accessories. For quantitative analysis of metabolites, integrals of resonances were determined and normalized to the number of cells and compared to the TSP standard (aqueous phase) or TMS standard (lipid phase) to obtain relative concentrations. Spectra were analyzed using the MNova software (Mestrelab Research, Santiago de Compostela, Spain).

### Clinical data collection

Molecular data from the TCGA TARGET GTEx database were retrieved from The UCSC Genome Browser database (RRID:SCR_005780, 10.1101/326470) [38]. Data were filtered selecting only samples from primary, treatment-naive tumors. The downloaded data included primary disease and gene expression (for Chk-α and PD-L1). No re-processing or re-normalization was performed on the data. The mean expression, standard deviation, and standard error of the mean were calculated for each tumor type. To investigate the correlation between the expression levels of Chk-α and PD-L1, we used the cBioPortal for cancer genomics (RRID:SCR_014555, https://www.cbioportal.org/) [39], using the messenger RNA (mRNA) expression *z*-scores (RNA Seq V2 RSEM) with a *z*-score threshold ± 2.0.

### Statistical analysis and reproducibility

Statistical analyses were performed using the GraphPad Prism 4 software (GraphPad Software, Inc., San Diego, CA, USA, RRID:SCR_002798). To determine the statistical significance of the quantified data, an unpaired two-tailed Student’s *t* test was performed. *p* values ≤ 0.05 were considered significant unless otherwise stated. To calculate the correlation between variables, the Agostino and Pearson normality test with an alpha value of 0.05 was used to test the normality of the data. If the data were normal, the Pearson correlation was used as previously described. If the data were not normal, the nonparametric Spearman correlation coefficient was used. All the statistical analyses were performed using two-tailed tests.

## Results

### Chk-α and PD-L1 expression are interactively related

**Fig. 1:**
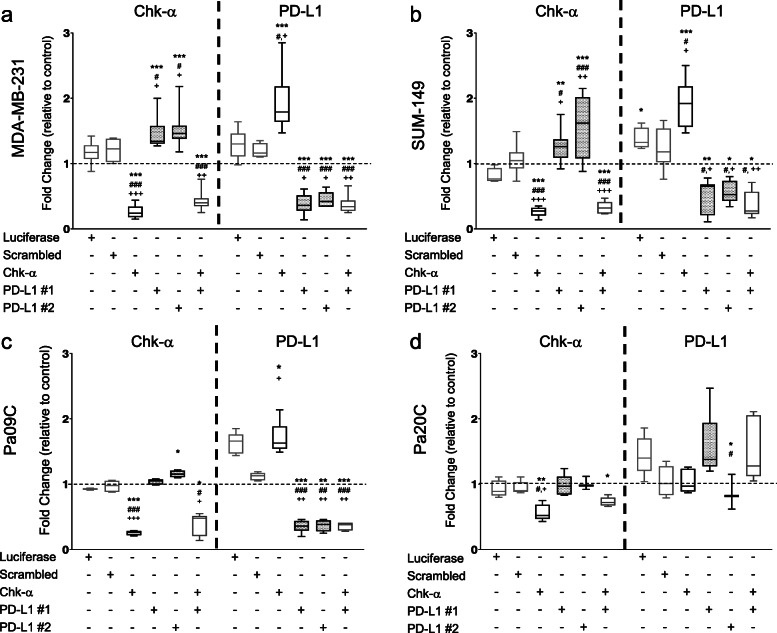
Chk-α and PD-L1 expression are interdependent. Relative fold change of Chk-α and PD-L1 mRNA expression in **a** MDA-MB-231, **b** SUM-149, **c** Pa09C, and **d** Pa20C cells. Fold changes were normalized to untreated cells as marked by the dotted line. Cells were transfected with 100 nM scrambled siRNA, 100 nM luciferase siRNA, 100 nM Chk-α siRNA, 100 nM PD-L1 siRNA #1, or 100 nM PD-L1 siRNA #2, or with a mixture of 50 nM PD-L1 #1 and 50 nM Chk-α siRNA. Values are presented as box and whisker plots, with the middle line representing the mean and the whiskers representing the maximum to minimum data points, from 5 to 15 independent experiments. Statistical significance was computed from the ∆C_t_ values. *p ≤ 0.05, **p ≤ 0.01, ***p ≤ 0.001, compared to untreated cells. ^#^*p* ≤ 0.05, ^##^*p* ≤ 0.01, ^###^*p* ≤ 0.001, compared to cells transfected with luciferase siRNA. ^+^*p* ≤ 0.05, ^++^*p* ≤ 0.01, ^+++^*p* ≤ 0.001, compared to cells transfected with scrambled siRNA (see also Supplementary Figure 1)

To identify the interactive relationship between Chk-α and PD-L1, we used siRNA to downregulate Chk-α and PD-L1 in TNBC MDA-MB-231 and SUM-149 cells, and in Pa09C and Pa20C human PDAC cells [32]. Untreated cells and cells treated with scrambled or luciferase siRNA were used as controls. We used a previously validated siRNA sequence [40] to downregulate Chk-α, and two separate siRNA sequences, labeled PD-L1 #1 and PD-L1 #2, to downregulate PD-L1. Changes in mRNA levels of Chk-α and PD-L1 in siRNA-treated cells, compared to untreated cells, are shown in Fig. 1. A significant reduction of Chk-α and PD-L1 mRNA levels was detected following treatment with the target-specific siRNAs, given singly or combined, in the two TNBC cell lines (Fig. 1a, b) and in Pa09C cells (Fig. 1c). In Pa20C cells, downregulation of Chk-α was less pronounced (< 50%), and there was no decrease in PD-L1 mRNA levels with PD-L1 siRNA treatment (Fig. 1d). Importantly, we found an inverse correlation between Chk-α and PD-L1 mRNA levels. Following Chk-α downregulation, PD-L1 increased by 85 ± 11% in MDA-MB-231, by 80 ± 15% in SUM-149, and by 70 ± 10% in Pa09C cells (values expressed as the mean ± standard error of the mean).

Conversely, in MDA-MB-231 and SUM-149 cells, a significant increase in Chk-α expression of more than 50% was observed following PD-L1 downregulation (Fig. 1a, b). In the PDAC cells, only Pa09C cells showed a small but significant increase in Chk-α mRNA expression with PD-L1 downregulation (Fig. 1c). Since PD-L1 was not downregulated in Pa20C cells, we did not detect an increase of Chk-α mRNA in these cells (Fig.1d). This inverse dependence was lost when cells were treated with a combination of Chk-αand PD-L1 siRNA. To ensure that the loss of inverse dependence was not due to the lower siRNA concentration, we treated MDA-MB-231 cells with 50 nM Chk-α or PD-L1#1 siRNA alone and observed similar changes as cells treated with 100 nM siRNA (data not shown). To further establish this inverse relationship, we analyzed the correlation between Chk-α and PD-L1 mRNA expression levels in all four cell lines treated with single siRNA (Supplementary Fig. 1). We found a strong inverse correlation in MDA-MB-231 (*p* < 0.0001, *r* = − 0.763) and SUM-149 (*p* < 0.0001, *r* = − 0.738) TNBC cells. Pa09C PDAC cells also showed a significant inverse correlation, although weaker than that observed in the TNBC cell lines (*p* = 0.001, *r* = − 0.676). Taken together, these data clearly identified an interdependence between Chk-α and PD-L1 at the genomic level. This interdependence was not observed when both genes were downregulated by siRNA.

### Changes in Chk-α and PD-L1 mRNA levels are reflected in protein changes

**Fig. 2:**
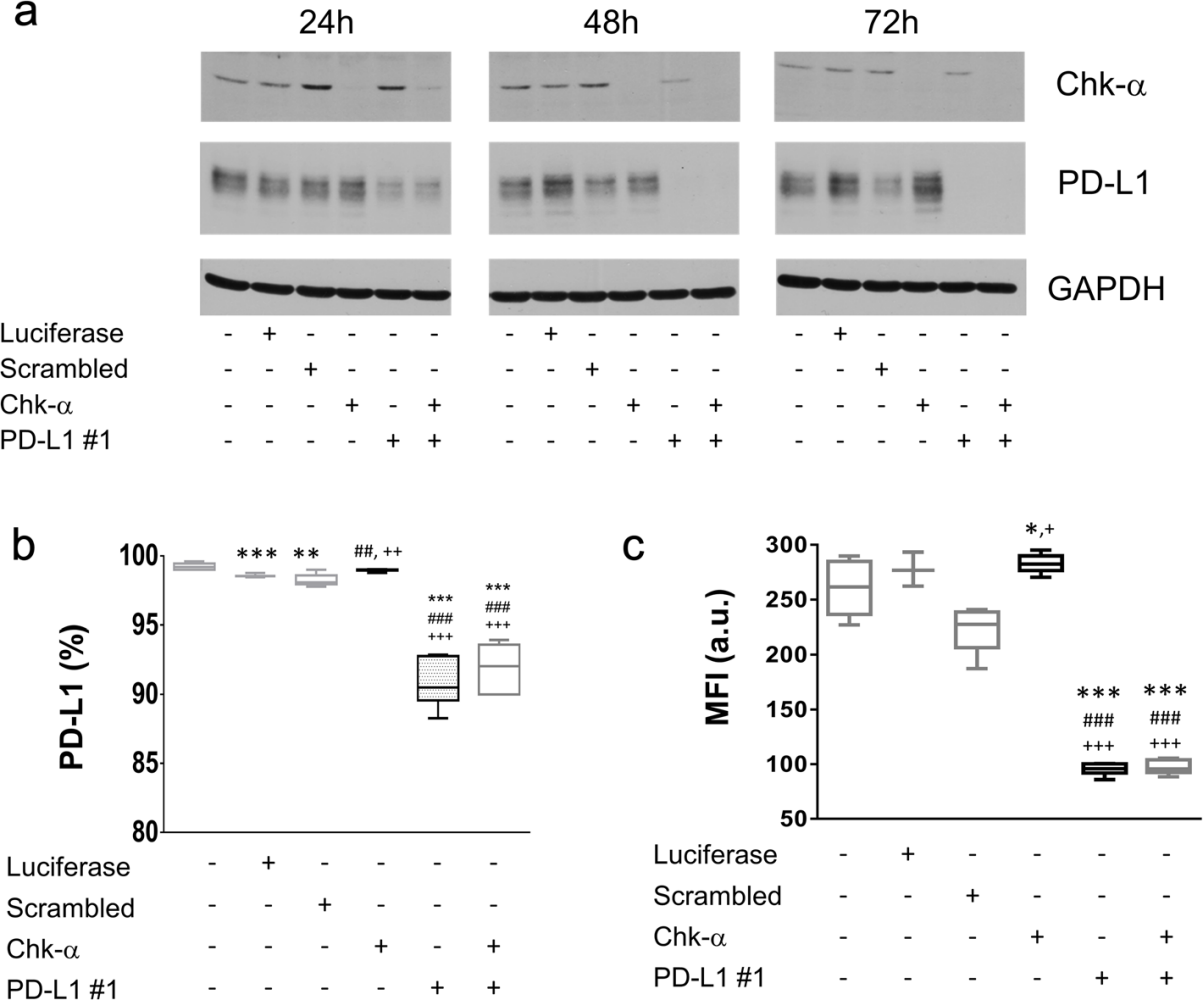
Changes in Chk-α and PD-L1 mRNA levels are reflected in the total protein levels. **a** Representative immunoblot assays of Chk-α (top), PD-L1 (middle), and GAPDH (bottom) at 24, 48, and 72 h of siRNA incubation, respectively. **b** Percentage of MDA-MB-231 cancer cells expressing PD-L1 protein on their surface measured by flow cytometry. **c** The mean fluorescence intensity (MFI) of PD-L1 in MDA-MB-231 cancer cells. MDA-MB-231 cells were transfected with 100 nM scrambled siRNA, 100 nM luciferase siRNA, 100 nM Chk-α siRNA, or 100 nM PD-L1 siRNA #1, or with a mixture of 50 nM PD-L1 #1 and 50 nM Chk-α siRNA. Values are presented as box and whisker plots, with the middle line representing the mean and the whiskers representing the maximum to minimum data points from 3 to 6 independent experiments. *p ≤ 0.05, **p ≤ 0.01, ***p ≤ 0.001, compared to untreated cells. ^**#**^***p*** ≤ 0.05, ^**##**^***p*** ≤ 0.01, ^**###**^***p*** ≤ 0.001, compared to cells transfected with luciferase siRNA. ^**+**^***p*** ≤ 0.05, ^**++**^***p*** ≤ 0.01, ^**+++**^***p*** ≤ 0.001, compared to cells transfected with scrambled siRNA (see also Supplementary Figure 2)

To determine whether changes in mRNA translated to changes in protein expression, protein levels of Chk-α and PD-L1 were measured in MDA-MB-231 cells by immunoblotting. As shown in Fig. 2a, proteins were harvested at 24 h, 48 h, and 72 h post-transfection with Chk-α and PD-L1 siRNA either alone or in combination. Chk-α and PD-L1 siRNA treatment resulted in an effective decrease of the targeted proteins when used alone or in combination. Immunoblots showed increased PD-L1 levels at 72 h following Chk-α siRNA treatment, in good agreement with previous studies that reported a delayed increase in PD-L1 protein levels compared with mRNA levels [41]. Chk-α increased at 24 h following PD-L1 siRNA treatment. This Chk-α/PD-L1 interdependence was not observed when cells were treated with both Chk-α and PD-L1 siRNA. Overall, the protein expression patterns were similar to the mRNA patterns.

To evaluate whether changes in PD-L1 mRNA and protein levels resulted in changes at the cell surface, we performed flow cytometry analysis of siRNA treated MDA-MB-231 cells (Fig. 2b, c and Supplementary Fig. 2). Treatment with PD-L1 #1 siRNA, either alone or in combination, was effective in reducing both the percentage of PD-L1-positive cells (Fig. 2b) and the mean fluorescence intensity (MFI, Fig. 2c), which reflects the total amount of PD-L1 on the cell surface. Treatment with Chk-α siRNA resulted in a small, but statistically significant increase in the percentage of PD-L1-positive cells when compared to cells treated with either scrambled or luciferase siRNA. This was also observed in MFI data when comparing Chk-α siRNA-treated cells with untreated or scrambled siRNA treated cells.

Taken together, these results confirmed that changes in mRNA resulted in changes in PD-L1 protein expression, and its localization on the cell surface. The magnitude of changes in PD-L1 cell surface expression was lower than anticipated based on the changes in mRNA and total level of proteins. This is likely due to the already high cell surface expression of PD-L1 in MDA-MB-231 cells, as reflected by the 99% of PD-L1-positive cells found in untreated cells, making any increase difficult to detect. However, a small but statistically significant increase was observed in the total amount of PD-L1 on the surface, as detected by MFI, in cells treated with Chk-α siRNA.

### Consequences of Chk-α and PD-L1 downregulation on metabolites

**Fig. 3:**
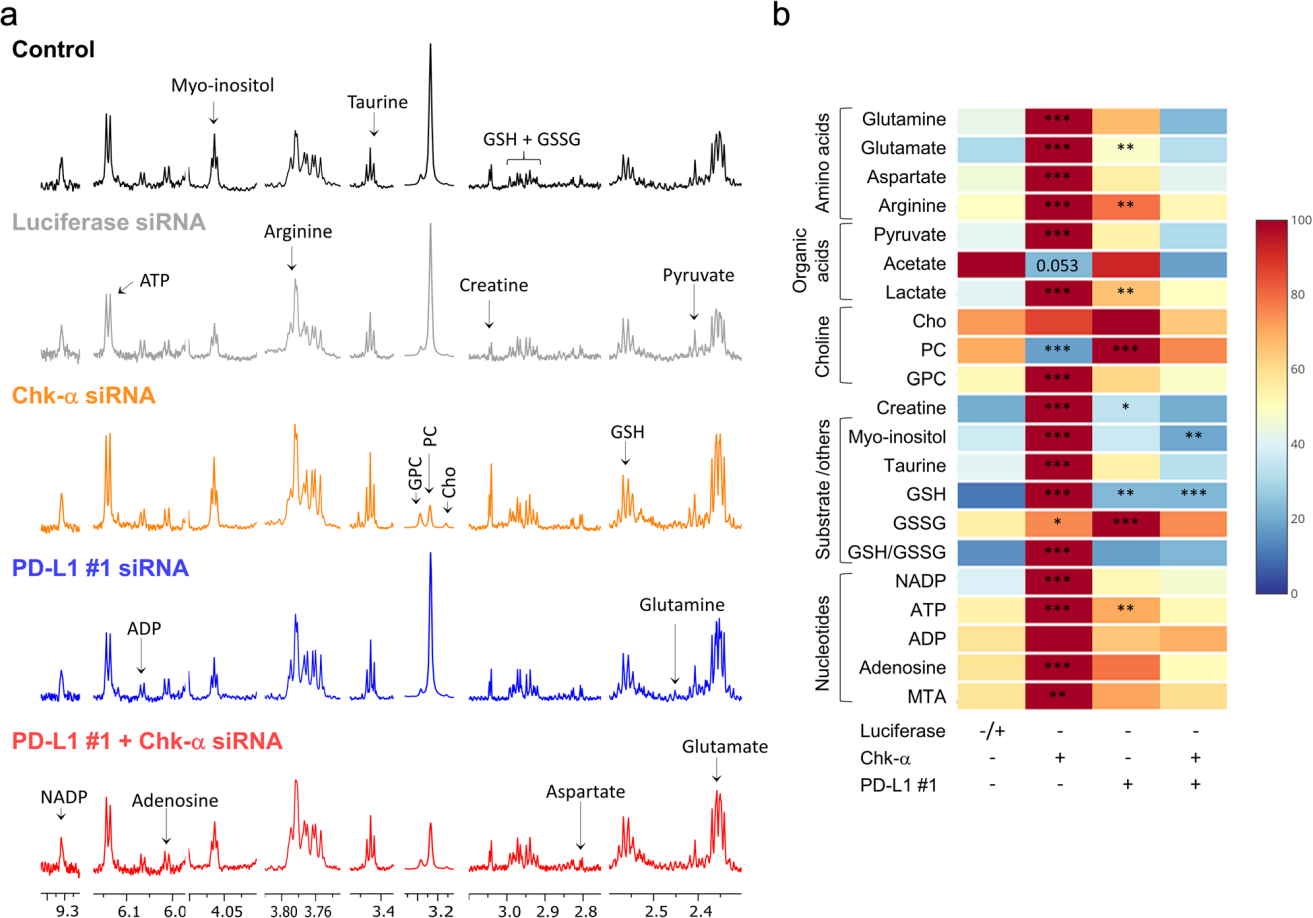
Consequences of Chk-α and PD-L1 downregulation on metabolites. **a** Representative high-resolution ^**1**^H MR spectra obtained from the aqueous phase of MDA-MB 231 cells. Spectra are displayed from untreated cells (black), cells transfected with 100 nM luciferase siRNA (light gray), cells transfected with 100 nM Chk-α siRNA (orange), cells transfected with 100 nM PD-L1 siRNA #1 (blue), and cells transfected with a mixture of 50 nM PD-L1 #1 and 50 nM Chk-α siRNA (red). All spectra were plotted on the same vertical scale and acquired with identical experimental parameters. GPC, glycerophosphocholine; PC, phosphocholine; Cho, choline; GSH, glutathione; GSSG, oxidized glutathione; MTA, ***S***-methyl-5′-thioadenosine. **b** Metabolic heat map, generated from quantitative analysis of high-resolution ^**1**^H MR spectral data of the aqueous phase, displaying differences in the metabolic profile of MDA-MB-231 cells. The heat map displays metabolites from untreated cells, cells transfected with 100 nM luciferase siRNA for 48 h, cells transfected with 100 nM Chk-α siRNA for 48 h, cells transfected with 100 nM PD-L1 siRNA #1 for 48 h, and cells transfected with a mixture of 50 nM PD-L1 #1 and 50 nM Chk-α siRNA for 48 h. Heat maps were created using the MATLAB software (MATLAB R2012b, MathWorks) to visualize the metabolic patterns. Due to the high dynamic range of metabolites, we normalized the highest intensity of a metabolite in each of the four groups to 100%. This normalization provides a dynamic range between 0 and 100%, allowing a better presentation of heat maps. The heat map represents the average of 3–6 replicates per group. The integral area under the peak was normalized to the number of cells for each sample. TSP dissolved in D_2_O was used as a quantitative reference in the spectral analysis. *p ≤ 0.05, **p ≤ 0.01, ***p ≤ 0.001, compared to the control group (see also Supplementary Table 1)

To assess whether changes in Chk-α or PD-L1 with siRNA treatment altered metabolites including choline-containing compounds, we used high-resolution ^1^H MRS to analyze the aqueous and lipid phases of MDA-MB-231 cell extracts. Significant differences in several metabolites were observed in MDA-MB-231 cells treated with PD-L1 or Chk-α siRNA individually, as shown in the representative aqueous phase spectra in Fig. 3a and the data summarized in Fig. 3b and Supplementary Table 1. These metabolic changes were mostly eliminated when both targets were downregulated. Data from untreated and luciferase siRNA-treated cells were combined into a single control group for a clearer presentation, since the metabolic profiles from these cells were comparable. With PD-L1 downregulation, a significant increase of PC was observed, consistent with the increase of Chk-α mRNA and protein found with PD-L1 downregulation. PD-L1 downregulation also resulted in a significant increase of glutamate, arginine, lactate, creatine, glutathione (GSH), oxidized glutathione (GSSG), and ATP. Treatment with Chk-α siRNA resulted in a significant decrease of PC demonstrating the functional effects of Chk-α downregulation that were in good agreement with previous results [42]. Chk-α downregulation resulted in a decrease of acetate, and a significant increase of glutamine, glutamate, aspartate, arginine, pyruvate, lactate, glycerophosphocholine (GPC), creatine, myo-inositol, taurine, GSH, GSSG, NADP, ATP, adenosine, and S-methyl-5′-thioadenosine (MTA). Finally, when both Chk-α and PD-L1 were downregulated together, most of these metabolic changes were not observed with the exception of changes in GSH and myo-inositol.

### Consequences of Chk-α and PD-L1 downregulation on lipids

**Fig. 4:**
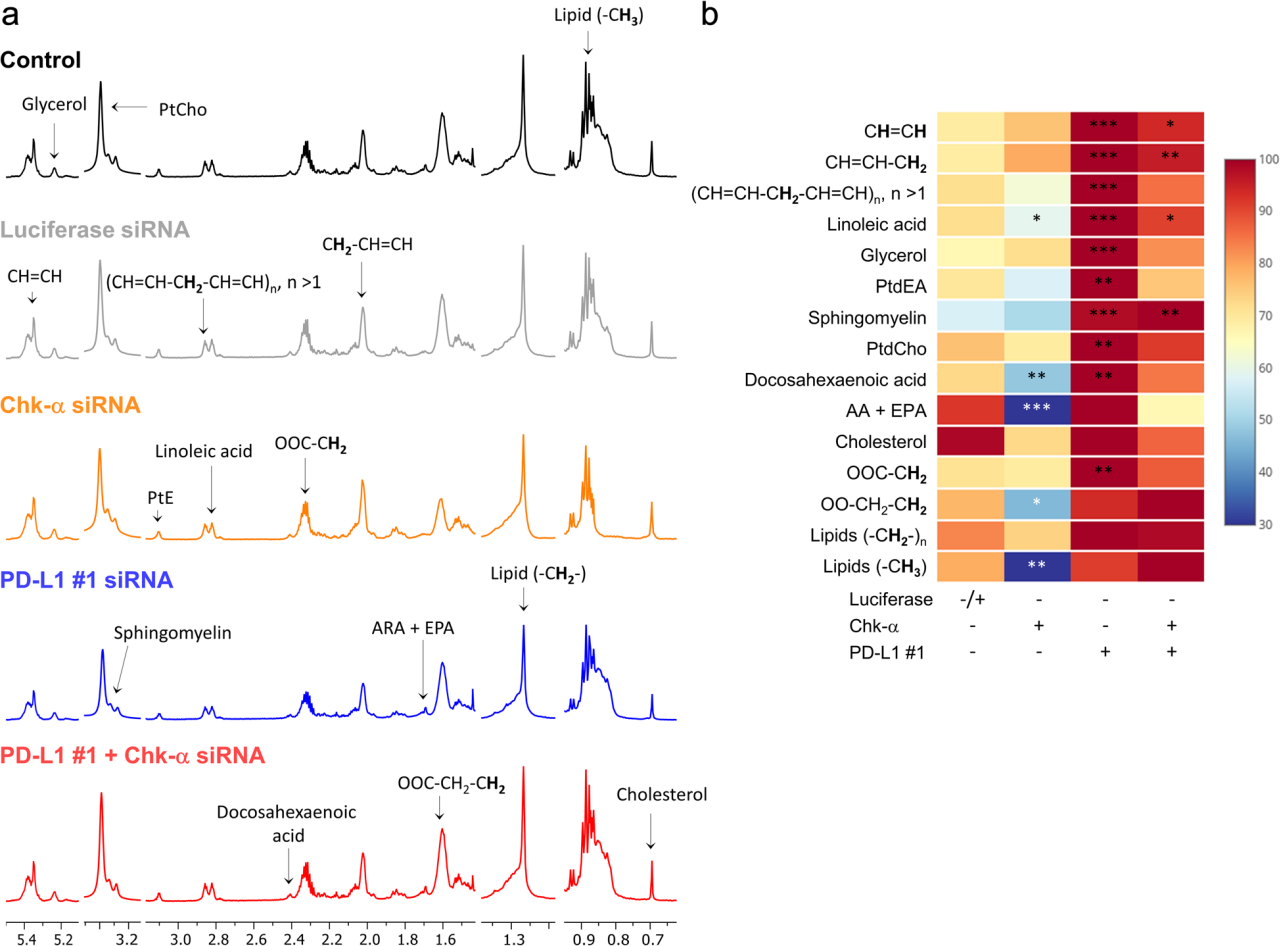
Consequences of Chk-α and PD-L1 downregulation on lipids. **a** Representative high-resolution ^**1**^H MR spectra obtained from the lipid phase of MDA-MB 231 cells. Spectra are displayed from untreated cells (black), cells transfected with 100 nM luciferase siRNA (light gray) for 48 h, cells transfected with 100 nM Chk-α siRNA (orange) for 48 h, cells transfected with 100 nM PD-L1 siRNA #1 (blue) for 48 h, and cells transfected with a mixture of 50 nM PD-L1 #1 and 50 nM Chk-α siRNA (red) for 48 h. **b** Metabolic heat map, generated from quantitative analysis of high-resolution ^**1**^H MR spectral data of the lipid phase, displaying differences in the lipid profile of MDA-MB-231 cells. The heat map displays metabolites from untreated cells, cells transfected with 100 nM luciferase siRNA for 48 h, cells transfected with 100 nM Chk-α siRNA for 48 h, cells transfected with 100 nM PD-L1 siRNA #1 for 48 h, and cells transfected with a mixture of 50 nM PD-L1 #1 and 50 nM Chk-α siRNA for 48 h. Heat maps were created as described above from 3 to 6 replicates per group. Lipids (-CH_3_), methyl groups of fatty acids; Lipids (-CH_2_-), methylene groups of fatty acids (truncated); OOC-CH_2_, methylene groups at the α position of the carboxylic function; OOC-CH_2_-CH_2_, methylene groups at the β position of the carboxylic function; ARA, arachidonic acid; EPA, eicosapentaenoic acid; PtdEA, phosphatidylethanolamine; PtdCholine, phosphatidylcholine; (CH=CH-CH_2_-CH=CH)_***n***_, diallylic methylene protons; CH=CH-CH_2_, methylene groups at the α position of a double bond; CH=CH, fatty acid double bonds. *p ≤ 0.05, **p ≤ 0.01, ***p ≤ 0.001, compared to the control group (see also Supplementary Table 2)

High-resolution ^1^H MRS of the lipid phase of MDA-MB-231 cell extracts detected significant changes in MRS detectable lipids following Chk-α or PD-L1 downregulation. Representative lipid phase spectra are shown in Fig. 4a with the changes in the lipid profile summarized in Fig. 4b and Supplementary Table 2. Untreated and luciferase siRNA-treated cells were combined into a single control group. Chk-α downregulation resulted in a significant decrease of the total lipid content, as represented by the methyl signal of fatty acids (-CH_3_) and the methylene groups at the β position of the carboxylic function (OOC-CH_2_-CH_2_). We also detected significantly decreased levels of arachidonic acid (AA) and eicosapentaenoic acid (EPA), docosahexaenoic acid, and linoleic acid. PD-L1 downregulation, consistent with the resultant increase of Chk-α, increased PtdCho, phosphatidylethanolamine (PtdEA), and the total level of lipids, as represented by the methylene groups at the α position of the carboxylic function (OOC-CH_2_). PD-L1 downregulation also caused an increase in the total level of unsaturated lipids, as represented by the fatty acid double bond signal (CH=CH), the methylene groups at the α position of a double bond (CH=CH-CH_2_) and diallylic methylene protons (CH=CH-CH_2_-CH=CH)_*n*_. We also detected significant increases in linoleic acid, glycerol, sphingomyelin, and docosahexaenoic acid. Changes in lipids induced with PD-L1 downregulation were mainly eliminated when both Chk-α and PD-L1 were downregulated, with the exception of sphingomyelin, linoleic acid, and unsaturated lipids as represented by CH=CH, and CH=CH-CH_2_, suggesting that these changes were mediated through Chk-α.

#### Inflammation and the Chk-α/PD-L1 interdependence

**Fig. 5:**
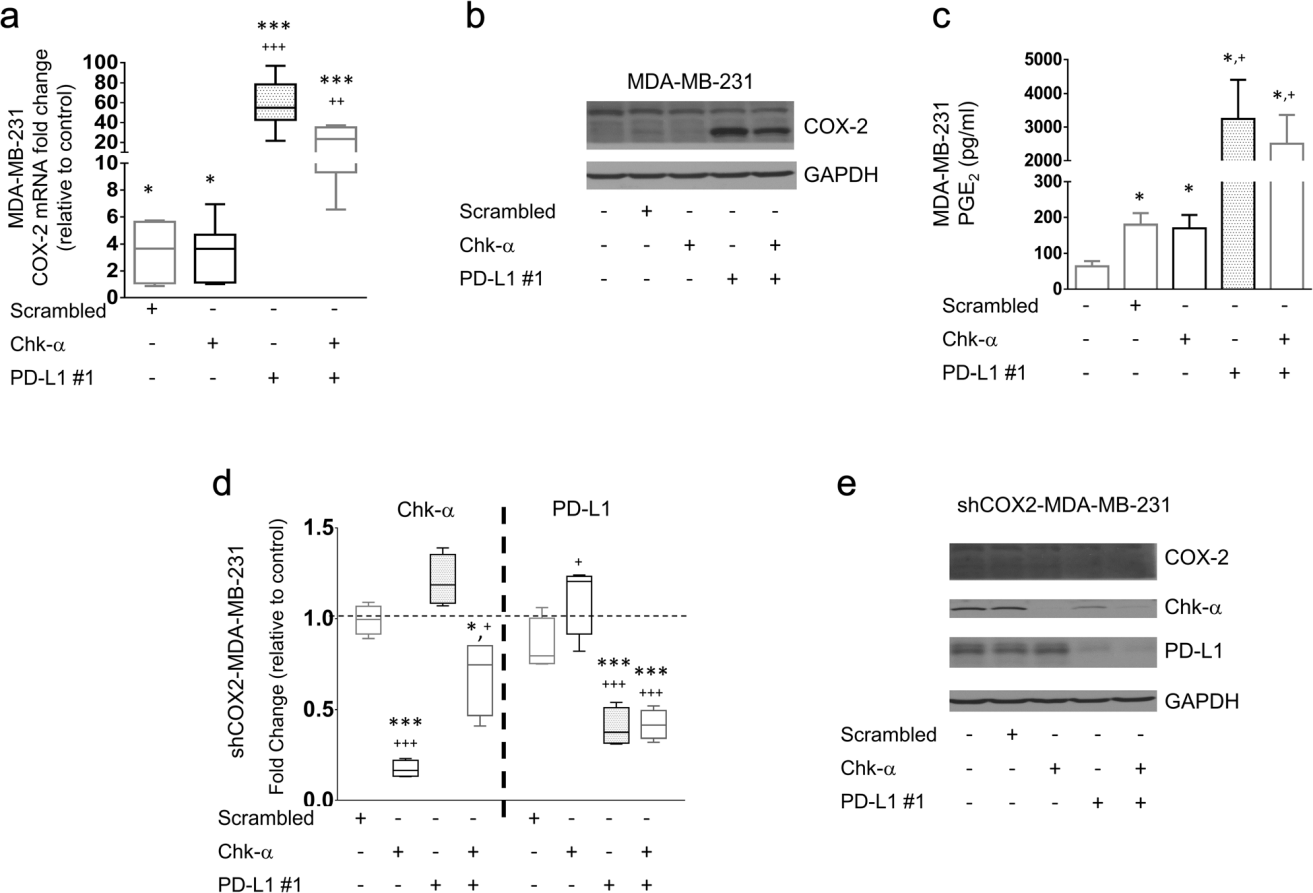
Role of cyclooxygenase 2 (COX-2) and prostaglandin E2 (PGE_2_) in the relationship between PD-L1 and Chk-α. **a** Changes of COX-2 mRNA expression levels, **b** protein levels, and **c** PGE_2_ production in the supernatant of MDA-MB-231 cells with different siRNA treatments. **d** Changes of Chk-α and PD-L1 mRNA expression levels at 48 h and **e** Chk-α and PD-L1 protein levels at 48 h of MDA-MB-231 cells with constitutive low expression of COX-2 (shCOX2-MDA-MB-231) with different siRNA treatments. siRNA treatments: untreated, transfected with 100 nM scrambled siRNA, transfected with 100 nM Chk-α siRNA, transfected with 100 nM PD-L1 siRNA #1, and transfected with a mixture of 50 nM PD-L1 #1 and 50 nM Chk-α siRNA. Data in **a** and **d** are presented as box and whisker plots, with the middle line representing the mean and the whiskers representing the maximum to minimum data points from 4 independent experiments. Values in **c** represent the mean ± SEM from 4 independent experiments. Statistical significance was calculated from ΔCt values. *p ≤ 0.05, **p ≤ 0.01, ***p ≤ 0.001, compared to untreated cells. ^+^*p* ≤ 0.05, ^++^*p* ≤ 0.01, ^+++^*p* ≤ 0.001, compared to cells transfected with scrambled siRNA

To further understand the mechanisms underlying the Chk-α/PD-L1 interdependence, we evaluated the role of inflammation and COX-2 in this relationship. COX-2 and its metabolite prostaglandin E2 (PGE_2_) play roles in inflammation, cancer development, and adaptation to changing microenvironments [43]. More recently, COX-2 and PGE_2_ have been implicated in cancer immunosuppression [44], and COX-2 and PD-L1 expressions were found to be correlated in melanomas [45] and lung adenocarcinomas [46]. Chk-α downregulation increased COX-2 mRNA expression by approximately 4-fold in MDA-MB-231 cells compared to untreated cells, but not compared to cells treated with scrambled siRNA (Fig. 5a). On the other hand, PD-L1 downregulation resulted in an over 40-fold increase of COX-2 mRNA, alone or in combination with Chk-α downregulation. Consistent with the relatively small increase of COX-2 with Chk-α downregulation, COX-2 protein levels (Fig. 5b) and PGE_2_ concentrations (Fig. 5c) did not increase with Chk-α siRNA treatment, whereas the 40-fold increase of COX-2 mRNA following treatment with PD-L1 siRNA resulted in an increase of COX-2 protein and PGE_2_ concentrations in the cell culture media. To further understand the role of COX-2 in the PD-L1/Chk-α dependence, we performed Chk-α and PD-L1 downregulation studies in MDA-MB-231 cells with COX-2 silenced using COX-2 short hairpin (sh)RNA (shCOX2-MDA-MB-231). We found that although Chk-α and PD-L1 siRNA downregulated the target genes, the increase of PD-L1 with Chk-α downregulation, and the increase of Chk-α with PD-L1 downregulation was eliminated in cells with COX-2 silenced, at the mRNA (Fig. 5d) and protein levels (Fig. 5e). These data indicate that COX-2 is required for the PD-L1 and Chk-α interdependence.

**Fig. 6:**
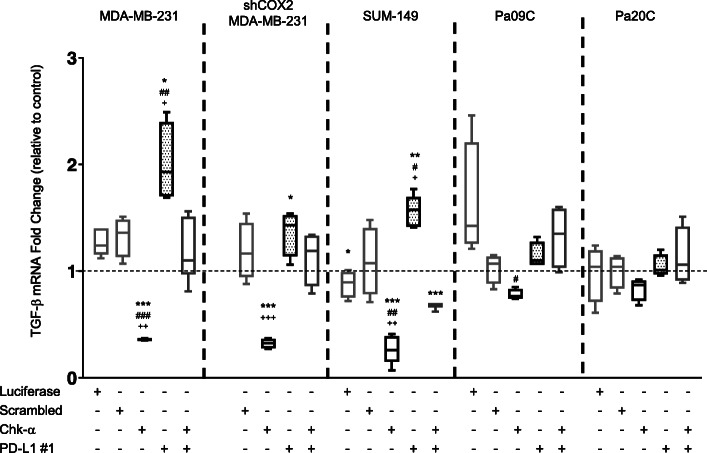
Role of transforming grow factor beta (TGF-β) in the relationship between PD-L1 and Chk-α. Relative fold change of TGF-β mRNA expression in MDA-MB-231, shCOX-2-MDA-MB-231, SUM-149, Pa09C, and Pa20C cells: untreated, transfected with 100 nM luciferase siRNA, transfected with 100 nM scrambled siRNA, transfected with 100 nM Chk-α siRNA, transfected with 100 nM PD-L1 siRNA #1, and transfected with a mixture of 50 nM PD-L1 and 50 nM Chk-α siRNA. Values presented as box and whisker plots, with the middle line representing the mean and the whiskers representing the maximum to minimum data points from 4 to 7 independent experiments. *p ≤ 0.05, **p ≤ 0.01, ***p ≤ 0.001, compared to untreated cells. ^#^*p* ≤ 0.05, ^##^*p* ≤ 0.01, ^###^*p* ≤ 0.001, compared to cells transfected with luciferase siRNA. ^+^*p* ≤ 0.05, ^++^*p* ≤ 0.01, ^+++^*p* ≤ 0.001, compared to cells transfected with scrambled siRNA

TGF-β, an inducer of COX-2 [47], has been directly associated with suppression of the host antitumor immune response [48] and resistance to immune therapies by increasing tumor cell plasticity [49]. We therefore analyzed changes in TGF-β expression in response to Chk-α and PD-L1 downregulation. As shown in Fig. 6, Chk-α downregulation significantly decreased TGF-β mRNA expression in TNBC MDA-MB-231, shCOX-2-MDA-MB-231, and SUM-149 cells. In PDAC cells, Pa09C showed a less pronounced decrease in TGF-β, while Pa20C showed the smallest decrease. These alterations matched the range of changes in the increase of PD-L1 observed with Chk-α downregulation, where TNBC cells showed the largest increase of PD-L1, followed by Pa09C cells, and Pa20C cells showed no increase. Conversely, downregulating PD-L1 resulted in an increase of TGF-β levels, that was most pronounced in TNBC, lesser in Pa09C cells, and none in Pa20C cells, matching the levels of PD-L1 downregulation and the corresponding increase of Chk-α. The increase of TGF-β in shCOX-2-MDA-MB-231 cells treated with PD-L1 siRNA was clearly attenuated compared to wild-type cells. When both Chk-α and PD-L1 were downregulated, no consistent pattern was observed.

### Chk-α and PD-L1 interdependence confirmed in human cancers

**Fig. 7:**
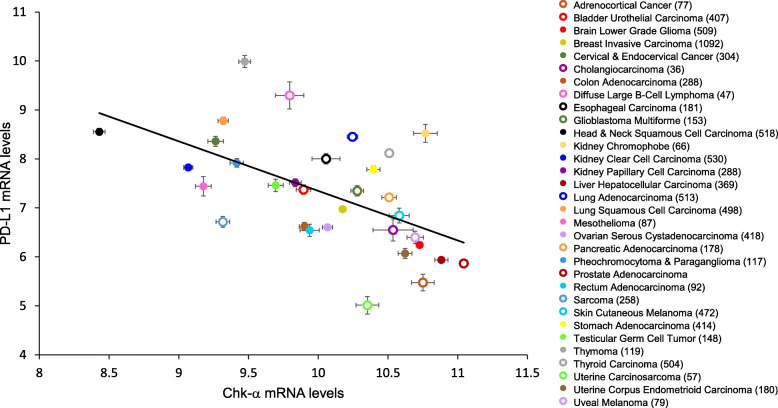
Correlation between the mean expression of Chk-α and PD-L1 in primary human cancers. The mean mRNA levels of Chk-α and PD-L1 in different tumor types showed a statistically significant correlation (*p* = 0.001, *r* = − 0.562) according to Pearson’s correlation coefficient. Data were obtained from the TCGA TARGET GTEx database. Numbers in parenthesis indicate the number of samples for each tumor type. Values are presented as dots, representing the mean value for each tumor type (color-coded), and the SEM for Chk-α (*X*-axis) and PD-L1 (*Y*-axis) (see also Supplementary Table 3, Supplementary Table 4, Supplementary Figure 3, and Supplementary Figure 4)

To independently confirm the inverse correlation between Chk-α and PD-L1 in human cancers, we analyzed the relationship between the tumoral expression of Chk-α and PD-L1 in TCGA. We calculated mean Chk-α and PD-L1 expression in primary tumor samples from 31 different cancer types (Supplementary Table 3), comprising of more than 9000 samples. We found an inverse linear correlation between PD-L1 and Chk-α mRNA levels (Fig. 7, *p* = 0.001, *r* = − 0.562). When we analyzed individual tumor values, irrespective of the tumor type (Supplementary Fig. 3), we also found a significant inverse correlation (*p* < 0.001, *r* = − 0.358). When we examined this relationship within individual tumor types, we found a statistically significant inverse correlation (*p* < 0.001) for 21 out of the 31 tumor types analyzed (Supplementary Table 4). When categorizing receptor status in the breast cancer group, we found that TNBC showed the most significant inverse correlation (*p* = 0.003, *r* = − 0.31), whereas the correlation was weaker or not statistically significant in the other breast cancers.

We further analyzed the TCGA samples by ranging primary tumors according to the mRNA levels of Chk-α and PD-L1 independently of the tumor type. Tumors with 10% lowest mRNA levels for Chk-α had significantly higher levels of PD-L1 (Supplementary Fig. 4A) compared with tumors with 10% highest mRNA levels for Chk-α. Similarly, tumors with 10% lowest mRNA levels for PD-L1 had significantly higher levels of Chk-α(Supplementary Fig. 4B) compared with tumors with 10% highest mRNA levels for PD-L1. These results are in good agreement with our experimental data showing an increase in PD-L1 with Chk-α downregulation and an increase in Chk-α with PD-L1 downregulation, further confirming this relationship in human cancers.

## Discussion

**Fig. 8:**
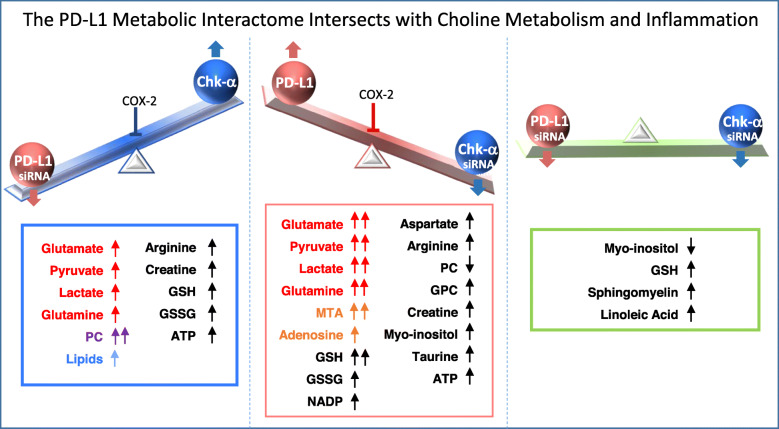
The PD-L1 metabolic interactome intersects with choline metabolism and inflammation. The inverse interdependence between PD-L1 and Chk-α together with the changes in metabolites with PD-L1 and Chk-α downregulation is summarized. Metabolites displayed in red are associated with PD-L1 regulation (glutamate, pyruvate, lactate, and glutamine), those in orange are related to creating an immune suppressive microenvironment (MTA, adenosine), those in purple are associated with escape from immune surveillance (PC) and lipids, highlighted in blue, skew tumor-infiltrating myeloid cells towards immunosuppressive and anti-inflammatory phenotypes

Our data identified previously unknown roles of the immune checkpoint PD-L1 in cancer cell metabolic reprogramming that are summarized in the schematic in Fig. 8. These data suggest that many of the metabolic changes are mediated through Chk-α, COX-2, and TGF-β. We found that PD-L1 expression significantly increased following downregulation of Chk-α, an enzyme that is overexpressed in most cancer cells. Conversely, downregulation of PD-L1 significantly increased the expression of Chk-α. This inverse dependence was eliminated when both genes were downregulated. Similarly, the metabolic changes detected with individual downregulation of Chk-α or PD-L1 were significantly attenuated when both genes were downregulated, identifying the interaction between these two molecules as necessary for many of the metabolic changes. The interaction between the two molecules was also eliminated in COX-2-silenced cells. TGF-β significantly decreased with Chk-α downregulation, significantly increased with PD-L1 downregulation, and remained unchanged when both Chk-α and PD-L1 were downregulated, identifying COX-2 and TGF-β as playing a role in this interactive relationship. The interaction between PD-L1 and Chk-α was observed in both breast cancer cell lines. However, one of the two PDAC cell lines, Pa20C, did not display this interactive relationship. Although one reason for the difference between the two PDAC cell lines may be that we were unable to achieve sufficient downregulation of Chk-α or PD-L1 in Pa20C cells to the levels achieved in Pa09C cells, the implications of these data on immune surveillance in different pancreatic cancer subtypes should also be considered. While the use of human cancer cells in immune-suppressed mice precludes evaluating changes in tumor immune cells in these xenograft models, techniques such as mass spectrometry imaging that allow overlay of metabolomic information with immunohistochemical analysis of immune cells in human cancer tissue may expand our understanding of the role of metabolism and the relationship between Chk-α and PD-L1 in immune surveillance.

Both Chk-α [17, 19, 50–53] and PD-L1 [54–59] play pro-oncogenic roles beyond their traditional functions in PC biosynthesis and in immunomodulation, respectively. Here, for the first time, we identified the direct involvement of Chk-α in immunosupression, as Chk-α downregulation significantly increased PD-L1 levels. Chk-α downregulation also shifted cancer cells towards a more immunosuppressive profile through metabolic reprogramming, increasing the production of metabolites such as lactate [12], glutamate [14], MTA [60], or glutamine [61], which have been linked to increased immune resistance of cancer cells. In a recent study, lower lactate production by cancer cells led to lower in vivo extracellular lactate and improved functioning of T cells [62]. Secretion of glutamate [63] and an excess of extracellular glutamate in the TME led to T cell dysfunction [64]. The increase of PD-L1 as a consequence of Chk-α downregulation, identified low Chk-α as contributing to immune suppression in cancer cells, different from its role as an oncogenic protein when overexpressed. Consequently, treatments that target Chk-α [27] may result in cancer cells escaping immune surveillance.

Downregulation of PD-L1 also resulted in a significant increase of immune-suppressive metabolites such as lactate [8, 62, 65], glutamate [14, 64], and PC [66], although the number of metabolites that were altered was fewer compared to Chk-α downregulation. The significant increase of lipid production and changes in the lipid profile following PD-L1 downregulation may also contribute to cancer cells escaping immune surveillance. Several studies have shown that lipids can reprogram tumor-infiltrating myeloid and T cells towards immunosuppressive and anti-inflammatory phenotypes [67–70]. The metabolic reprogramming observed following downregulation of PD-L1 and its effects on the TIME merit further investigation. This metabolic reprogramming that, based on our data, is mediated through Chk-α, may assist cancer cells in escaping immune surveillance in response to a decrease of PD-L1, or may be a component of the PD-L1 immune checkpoint program in activating the immune system. Further studies investigating metabolic reprogramming controlled by the different immune checkpoints will provide a more comprehensive understanding of the interaction between the immune checkpoints and metabolism.

PD-L1 downregulation may also create a more immunosuppressive profile through increased expression of TGF-β and COX-2, both of which are related to immune escape [71] and anti-PD-L1 treatment failure [72]. We detected increased TGF-β, COX-2, and lipid production with PD-L1 downregulation. Conversely, TGF-β and lipid production decreased following increased PD-L1 expression in response to Chk-α downregulation. When both Chk-α and PD-L1 were downregulated, or when COX-2 was silenced, these changes were eliminated. Our data support an active role of both TGF-β and COX-2 in the dependence between Chk-α and PD-L1. Although not investigated here, the roles of the inflammatory transcription factor NF-kappa-B and HIF regulation in mediating the interactions between Chk-α and PD-L1 should be investigated in future studies.

COX-2 inhibition downregulated PD-L1 levels in Lewis lung carcinoma and CMT167 models [73] and in human melanoma cells [45]. On the other hand, COX-2 inhibition did not impact PD-L1 levels of lung cancer cells [46], or only affected tumor-associated macrophages and myeloid-derived suppressor cells [41], or decreased PD-L1 by a COX-2/PGE_2_ independent pathway in breast cancer cells [74]. Our data are consistent with previous observations of TGF-β inversely correlating with PD-L1 expression in neuroblastoma cells [75]. Furthermore, several preclinical studies have shown that targeting the TGF-β signaling pathway synergizes with PD-L1 blocking, improving tumor control and enhancing anti-tumor immunity [72, 76]. Clinically, it is well established that different tumor types use TGF-β production to evade immune attack [71], and the overexpression of TGF-β and PGE_2_ diminished tumor recognition by T cells [77]. Our results demonstrate, for the first time, that PD-L1 plays a significant role in COX-2 and TGF-β modulation in cancer cells.

A major unmet need in treatment with immune checkpoint inhibitors is the lack of a noninvasive technique to identify patients who may benefit from such a therapy [78]. Our results suggest that tumors with low PD-L1 expression may have high Chk-α expression and consequently high PC and total choline that can be detected noninvasively with ^1^H MRS. Future studies relating total choline detected by ^1^H MRS in tumors to PD-L1 expression in biopsy samples may provide further evidence for the development of the total choline signal as a biomarker to predict for PD-L1 expression levels [16, 24].

## Conclusions

Although the role of immune checkpoints in immune cell metabolism has been widely studied [6], few data are available on the regulation of cancer cell metabolism by immune checkpoints. Immune checkpoint overexpression has been linked to increased glycolysis and lactate production in breast cancer cells both in vitro and in vivo [79]. Targeting protein B7-H3 (CD276) in cancer cells at the genomic level [79] or PD-L1 with antibodies in a sarcoma model [56], resulted in decreased glycolysis and glucose consumption both in vitro and in vivo. We identified for the first time, a dual role for PD-L1, as being modulated by and being a modulator of tumor metabolism. Similarly, we identified for the first time the role of Chk-α in immunosuppression. This is especially relevant as this gene is commonly overexpressed in most cancers [16]. These observations may provide new insights in the rational design of combinatorial therapies targeting immune checkpoint inhibitors and cancer metabolism.

## Supplementary Information

**Additional file 1: Supplementary Figure 1.** Correlation between the mean expression of Chk-α and PD-L1 in different cancer cell lines when treated with siRNA for 48h corresponding to Figure 1. Plot showing a correlation between mRNA expression level of Chk-α and PD-L1 obtained by RT-PCR of (**A**) MDA-MB-231, (**B**) SUM 149, (**C**) Pa09C and (**D**) Pa20C cells. Statistical analysis using Pearson’s correlation coefficient showed a significant correlation with P≤0.001 for all except Pa20C cells. **Supplementary Figure 2.** Representative flow cytometry histograms for MDA-MB-231 cells treated with siRNA corresponding to Figure 2. Representative flow cytometry histograms showing signals from control IgG-APC (blue) and anti-PD-L1-APC (red) antibodies in MDA-MB-231 cells: untreated (**A**), transfected with 100 nM scrambled siRNA (**B**), transfected with 100 nM luciferase siRNA (**C**), transfected with 100 nM Chk-siRNA (**D**), transfected with 100 nM PD-L1 siRNA (**E**) and transfected with a mixture of 50 nM PD-L1 and 50 nM Chk-α siRNA (Chk-α + PD-L1) (**F**). **Supplementary Figure 3.** Correlation between the expression of Chk-α and PD-L1 in primary tumor tissue among different human cancers corresponding to Figure 7. Individual levels of Chk-α and PD-L1 measured in different tumor types showed a statistically significant correlation (*P*< 0.001, r=-0.358) according to Spearman's correlation coefficient. **Supplementary Figure 4.** Comparison between Chk-α and PD-L1 in primary tumors with the highest and lowest values of these genes corresponding to Figure 7. We ranged primary tumors from the TCGA TARGET GTEx database according to their mRNA levels for Chk-α and PD-L1. We selected those samples, irrespective of the tumor type, based on the 10% highest and 10% lowest values for (**A**) Chk-α and (**B**) PD-L1. **Supplementary Table 1.** Mean values of water-soluble metabolite concentrations in MDA-MB-231 cells corresponding to Figure 3. Values were generated from the quantitative analysis of high-resolution ^1^H MR spectra obtained at 48h from the aqueous phase of MDA-MB-231 cells that were: untreated, transfected with 100 nM luciferase siRNA (Luciferase), transfected with 100 nM Chk-α siRNA (Chk-α), transfected with 100 nM PD-L1 #1 siRNA (PD-L1) and transfected with a mixture of 50 nM PD-L1 and 50 nM Chk-α siRNA (Chk-α + PD-L1). Values represent Mean (mM /cell) ± SEM from 3-6 independent experiments. GPC: glycerophosphocholine, PC: phosphocholine, Cho: choline, GSH: glutathione, GSSG: oxidized glutathione, MTA: S-methyl-5′-thioadenosine. **Supplementary Table 2.** Mean values of lipid metabolites in MDA-MB-231 cells corresponding to Figure 4. Values were generated from the quantitative analysis of high-resolution ^1^H MR spectra obtained at 48h from the lipid phase of MDA-MB-231 that were: untreated (control), transfected with 100 nM luciferase siRNA (Luciferase), transfected with 100 nM Chk-α siRNA (Chk-α), transfected with 100 nM PD-L1 #1 siRNA (PD-L1) and transfected with a mixture of 50 nM PD-L1 and 50 nM Chk-α siRNA (Chk-α + PD-L1). Values represent Mean (a.u.) ± SEM obtained from 3-6 independent experiments. Lipids (-C**H**_**3**_): methyl groups of fatty acids, Lipids (-C**H**_**2**_-): methylene groups of fatty acids, OOC-C**H**_**2**_: methylene groups at the α position of the carboxylic function, OOC-CH_2_-C**H**_**2**_: methylene groups at the β position of the carboxylic function, ARA: arachidonic acid, EPA: eicosapentaenoic acid, PtdEA: phosphatidylethanolamine, PtdCholine: phosphatidylcholine, (CH=CH-C**H**_**2**_-CH=CH)_n_: diallylic methylene protons, CH=CH-C**H**_**2**_: methylene groups at the α position of a double bond, C**H**=C**H**: fatty acid double bonds. **Supplementary Table 3.** Mean values for Chk-α and PD-L1 expression for 32 different tumor types corresponding to Figure 7. Values were extracted from the TCGA public database and expressed as the Mean ± SEM. The number of tumor samples for each tumor type available in the TCGA data base are also presented. **Supplementary Table 4.** Correlation coefficients between PD-L1 and Chk-α expression in different tumor types corresponding to Figure 7. Correlation coefficients were calculated used the c-bioportal, and by selecting mRNA Expression Z-scores (RNA Seq V2 RSEM) with a z-score threshold of ±2.0. Statistically significant correlations (p<0.01) are highlighted in bold.

## Data Availability

Lead contact: Further information and requests for resources and reagents should be directed to and will be fulfilled by the lead contact, Zaver M Bhujwalla (zbhujwa1@jhmi.edu). Data availability: The accession number for the raw spectra data reported in this paper is DOI: 10.17632/r4z94fxtwx.1. Data are uploaded into Mendeley Data. The published article (in supplementary information) includes all TCGA data analyzed during this study (Supplementary Tables 3 and 4).

## Abbreviations

AA: Arachidonic acid
Chk-α: Choline kinase-α
Cho: Choline
COX-2: Prostaglandin-endoperoxide synthase 2
EPA: Eicosapentaenoic acid
GSH: Glutathione
GSSG: Oxidized glutathione
GPC: Glycerophosphocholine
HPRT1: Hypoxanthine phosphoribosyltransferase 1
MFI: Mean fluorescence intensity
mRNA: Messenger RNA
MRS: Magnetic resonance spectroscopy
MTA: *S*-methyl-5′-thioadenosine
PD-1: Programmed cell death protein-1
PD-L1: Programmed death-ligand 1
PDAC: Human pancreatic ductal adenocarcinoma
TCGA: The Cancer Genome Atlas Program
TIME: Tumor immune microenvironment
TME: Tumor microenvironment
TNBC: Human triple-negative breast cancer
PC: Phosphocholine
PGE_2_: Prostaglandin E2
PtdCho: Phosphatidylcholine
PtdEA: Phosphatidylethanolamine
rt-PCR: Real-time polymerase chain reaction
shRNA: Short hairpin RNA
siRNA: Small interfering RNA
STR: Short tandem repeat
TGF-β: Transforming growth factor beta
TMS: Tetramethylsilane
TSP: 3-(Trimethylsilyl) propionic 2,2,3,3-d4 acid sodium salt
