# Supplementary material for: The PD-L1 metabolic interactome intersects with choline metabolism and inflammation
Source: Cancer Metab. 2021 Feb 19;9:10. doi: 10.1186/s40170-021-00245-w (PMC7893974; doi:10.1186/s40170-021-00245-w)
Supplement: Supplementary file 1 — Additional file 1: Supplementary Figure 1. Correlation between the mean expression of Chk-α and PD-L1 in different cancer cell lines when treated with siRNA for 48h corresponding to Figure 1. Plot showing a correlation between mRNA expression level of Chk-α and PD-L1 obtained by RT-PCR of (A) MDA-MB-231, (B) SUM 149, (C) Pa09C and (D) Pa20C cells. Statistical analysis using Pearson’s correlation coefficient showed a significant correlation with P≤0.001 for all except Pa20C cells. Supplementary Figure 2. Representative flow cytometry histograms for MDA-MB-231 cells treated with siRNA corresponding to Figure 2. Representative flow cytometry histograms showing signals from control IgG-APC (blue) and anti-PD-L1-APC (red) antibodies in MDA-MB-231 cells: untreated (A), transfected with 100 nM scrambled siRNA (B), transfected with 100 nM luciferase siRNA (C), transfected with 100 nM Chk-siRNA (D), transfected with 100 nM PD-L1 siRNA (E) and transfected with a mixture of 50 nM PD-L1 and 50 nM Chk-α siRNA (Chk-α + PD-L1) (F). Supplementary Figure 3. Correlation between the expression of Chk-α and PD-L1 in primary tumor tissue among different human cancers corresponding to Figure 7. Individual levels of Chk-α and PD-L1 measured in different tumor types showed a statistically significant correlation (P< 0.001, r=-0.358) according to Spearman's correlation coefficient. Supplementary Figure 4. Comparison between Chk-α and PD-L1 in primary tumors with the highest and lowest values of these genes corresponding to Figure 7. We ranged primary tumors from the TCGA TARGET GTEx database according to their mRNA levels for Chk-α and PD-L1. We selected those samples, irrespective of the tumor type, based on the 10% highest and 10% lowest values for (A) Chk-α and (B) PD-L1. Supplementary Table 1. Mean values of water-soluble metabolite concentrations in MDA-MB-231 cells corresponding to Figure 3. Values were generated from the quantitative analysis of high-resolution 1H MR spectra obtained [file 40170_2021_245_MOESM1_ESM.pdf]

Supplementary Figures. Titles and Legends.

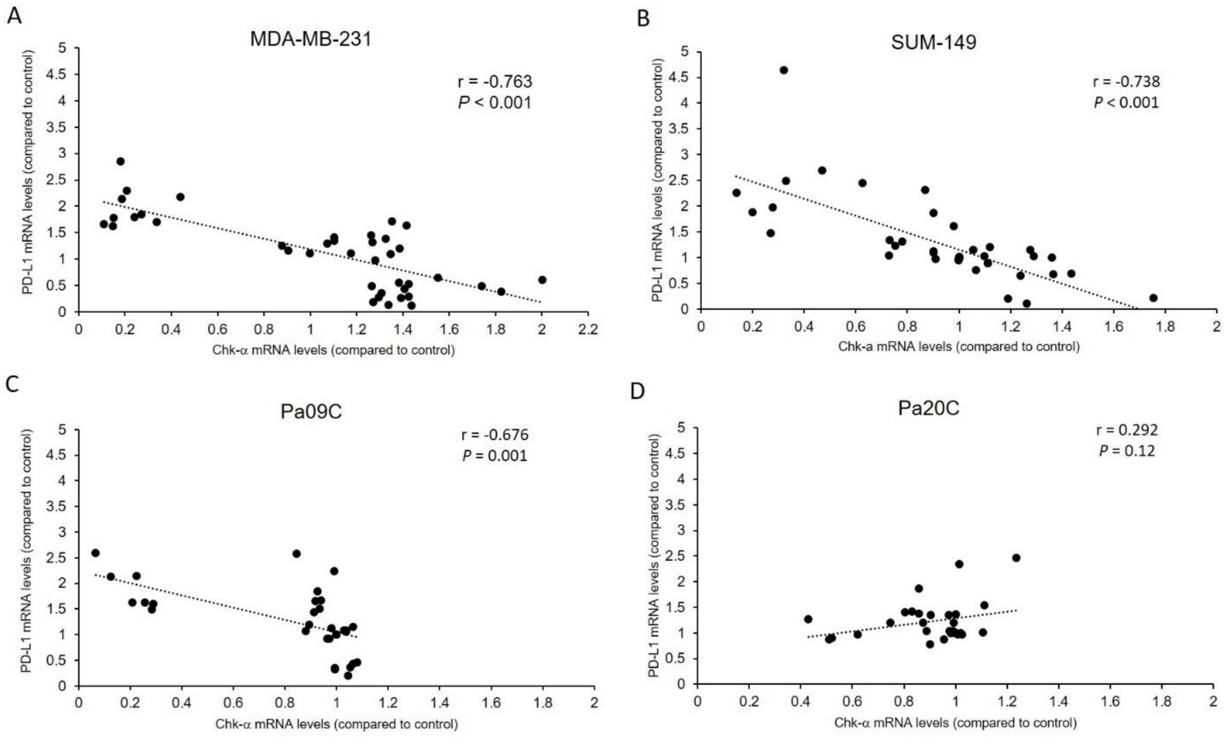

Supplementary Figure 1. **Correlation between the mean expression of Chk-α and PD-L1 in different cancer cell lines when treated with siRNA for 48h, related to Figure 1.**

Plot showing a correlation between mRNA expression level of Chk-α and PD-L1 obtained by RT-PCR of (**A**) MDA-MB-231, (**B**) SUM 149, (**C**) Pa09C and (**D**) Pa20C cells. Statistical analysis using Pearson's correlation coefficient showed a significant correlation with P≤0.001 for all except Pa20C cells.

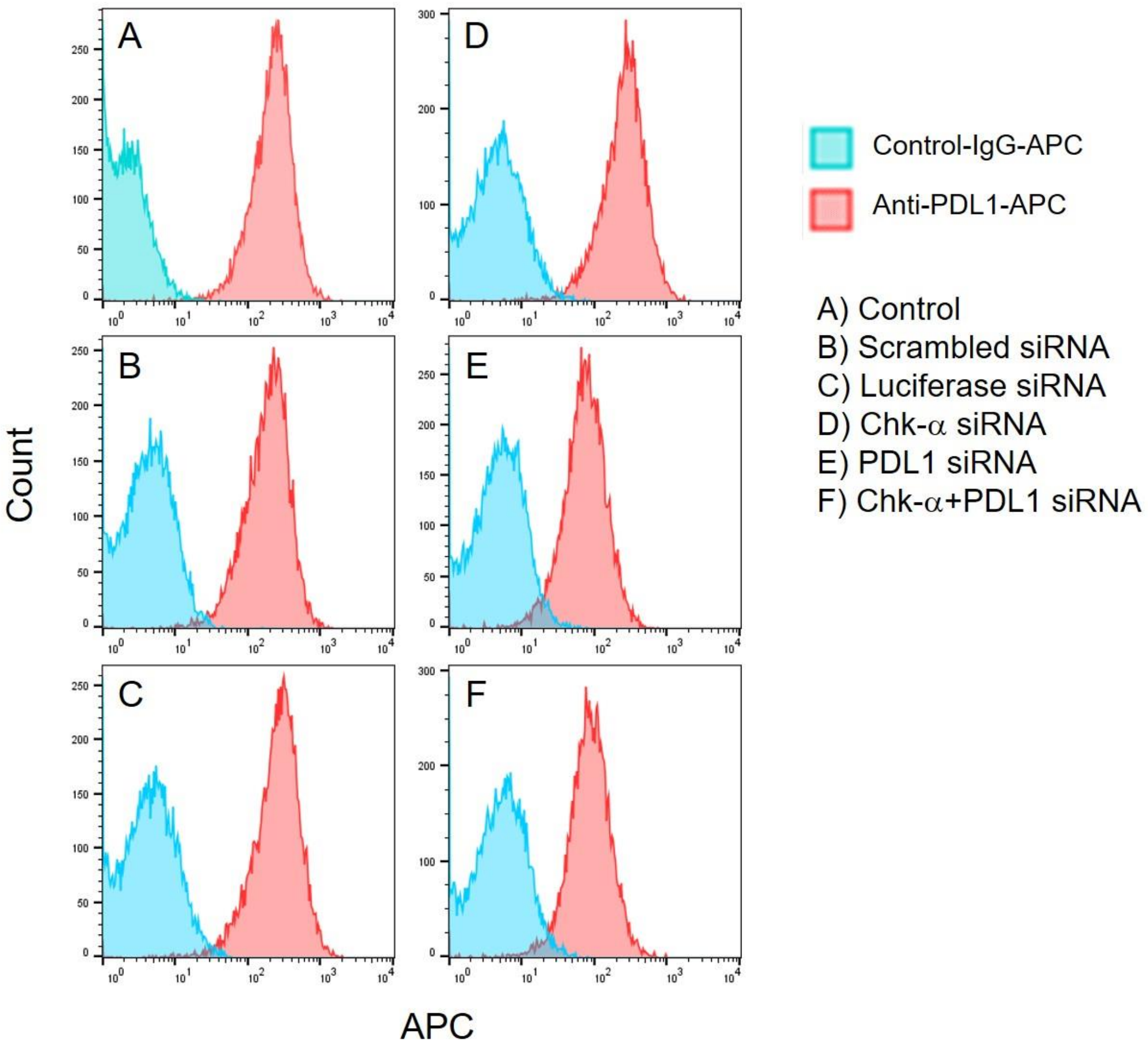

Supplementary Figure 2. Representative flow cytometry histograms for MDA-MB-231 cells treated with siRNA, related to Figure 2.

Representative flow cytometry histograms showing signals from control IgG-APC (blue) and antiPD-L1-APC (red) antibodies in MDA-MB-231 cells untreated (**A**), treated with scrambled siRNA (**B**), luciferase siRNA (**C**), Chk-siRNA (**D**), PD-L1 siRNA (**E**) and Chk+PD-L1 siRNA (**F**).

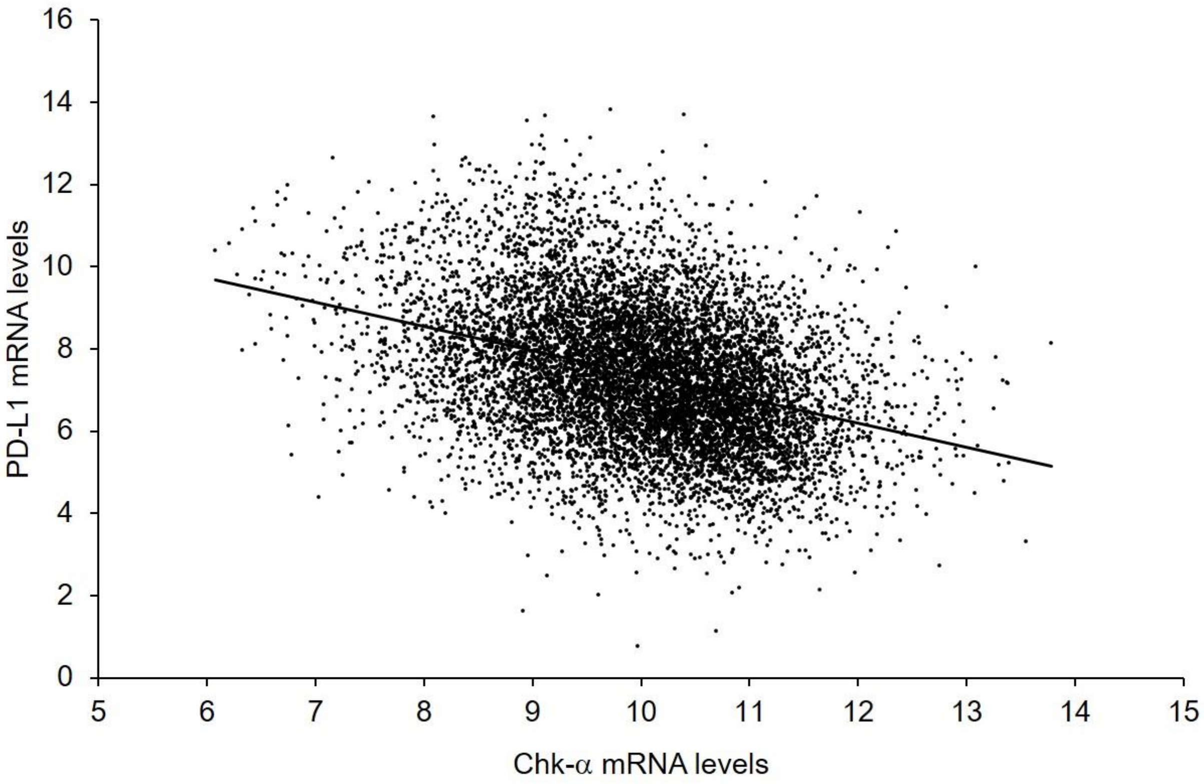

Supplementary Figure 3. Correlation between the expression of Chk-α and PD-L1 in primary tumor tissue among different human cancers, related to Figure 7.

Individual levels of Chk-α and PD-L1 measured in different tumor types showed a statistically significant correlation (*P*< 0.001, r=-0.358) according to Spearman's correlation coefficient.

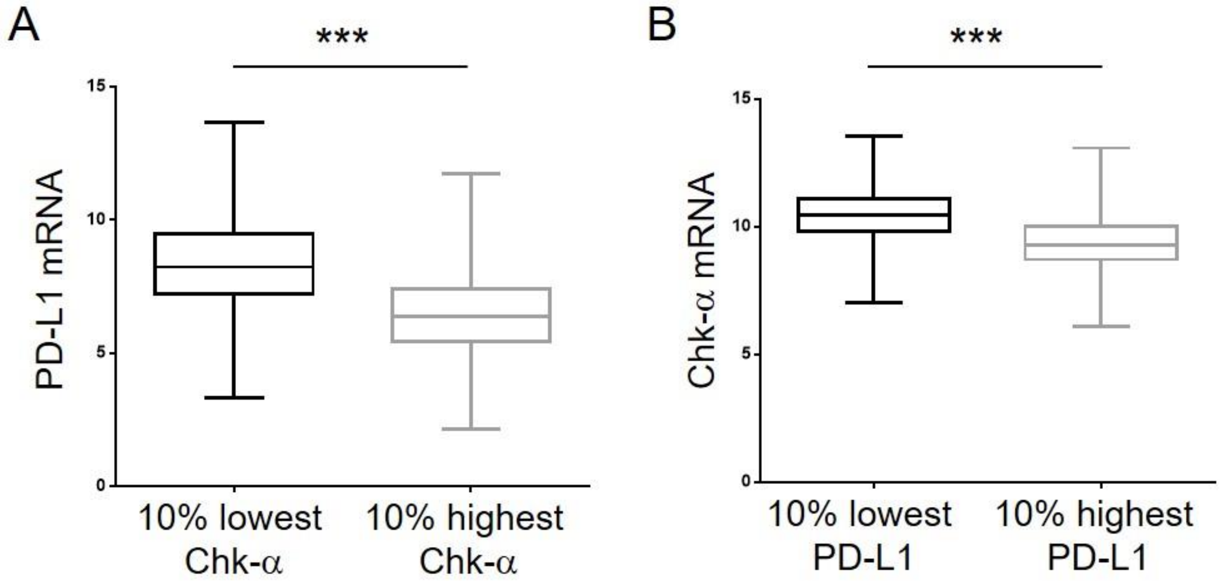

Supplementary Figure 4. Comparison between Chk-α and PD-L1 in primary tumors with the highest and lowest values of these genes, related to Figure 7.

We ranged primary tumors from the TCGA TARGET GTEx database according to their mRNA levels for Chk-α and PD-L1. We selected those samples, irrespective of the tumor type, based on the 10% highest and 10% lowest values for (**A**) Chk-α and (**B**) PD-L1.

Supplementary Tables. Titles and Legends.

	Untreated	Luciferase	Chk-α	PD-L1	Chk-α + PD-L1
Glutamine	0.09 ± 0.04	0.17 ± 0.02	0.27 ± 0.04	0.21 ± 0.04	0.06 ± 0.02
Glutamate	1.4 ± 0.2	1.7 ± 0.2	4.9 ± 0.2	2.8 ± 0.5	1.6 ± 0.1
Aspartate	0.33 ± 0.07	0.47 ± 0.08	0.84 ± 0.09	0.53 ± 0.09	0.35 ± 0.06
Arginine	2.3 ± 0.2	3.3 ± 0.3	5.5 ± 0.4	4.5 ± 0.4	2.9 ± 0.2
Pyruvate	0.08 ± 0.02	0.12 ± 0.01	0.23 ± 0.03	0.15 ± 0.03	0.070 ± 0.003
Acetate	1.1 ± 0.6	3.1 ± 0.5	0.42 ± 0.02	2.1 ± 0.7	0.34 ± 0.02
Lactate	1.5 ± 0.3	2.5 ± 0.3	4.6 ± 0.3	3.3 ± 0.2	2.3 ± 0.1
Cho	0.16 ± 0.05	0.13 ± 0.01	0.17 ± 0.01	0.22 ± 0.03	0.13 ± 0.03
PC	3.0 ± 0.2	3.5 ± 0.4	0.81 ± 0.05	4.8 ± 0.3	3.4 ± 0.3
GPC	0.30 ± 0.03	0.35 ± 0.03	0.62 ± 0.03	0.39 ± 0.03	0.30 ± 0.01
Creatine	0.08 ± 0.02	0.12 ± 0.03	0.46 ± 0.02	0.16 ± 0.02	0.09 ± 0.02
Myo-inositol	1.0 ± 0.1	0.86 ± 0.09	2.6 ± 0.1	1.1 ± 0.2	0.50 ± 0.07
Taurine	0.98 ± 0.08	1.8 ± 0.3	3.1 ± 0.2	1.8 ± 0.2	1.0 ± 0.1
GSH	0.45 ± 0.02	0.63 ± 0.06	4.8 ± 0.6	1.3 ± 0.2	1.07 ± 0.09
GSSG	0.14 ± 0.01	0.17 ± 0.03	0.21 ± 0.02	0.29 ± 0.03	0.21 ± 0.02
GSH/GSSG	3.3 ± 0.4	4.0 ± 0.9	24.2 ± 3.2	4.5 ± 0.8	5.4 ± 0.8
NADP	0.07 ± 0.01	0.09 ± 0.02	0.21 ± 0.02	0.13 ± 0.02	0.10 ± 0.01
ATP	1.3 ± 0.1	1.6 ± 0.2	2.67 ± 0.11	2.0 ± 0.2	1.39 ± 0.08
ADP	0.14 ± 0.01	0.56 ± 0.03	0.51 ± 0.08	0.36 ± 0.07	0.36 ± 0.05
Adenosine	1.7 ± 0.2	2.3 ± 0.2	3.6 ± 0.3	2.9 ± 0.3	2.0 ± 0.1
MTA	0.20 ± 0.03	0.25 ± 0.03	0.37 ± 0.03	0.29 ± 0.04	0.224 ± 0.003

Supplementary Table 1. Mean values of water-soluble metabolite concentrations in MDA-MB-231 cells, related to Figure 3.

Values were generated from the quantitative analysis of high-resolution ^{1}H MR spectra obtained at 48 h from the aqueous phase of MDA-MB-231 cells that were: untreated, transfected with 100 nM luciferase siRNA (Luciferase), transfected with 100 nM Chk-α siRNA (Chk-α), transfected with 100 nM PD-L1 #1 siRNA (PD-L1) and transfected with a mixture of 50 nM PD-L1 and 50 nM Chk-α siRNA(Chk-α + PD-L1). Values represent Mean (mM /cell) ± SEM from 3-6 independent experiments.

GPC: glycerophosphocholine, PC: phosphocholine, Cho: choline, GSH: glutathione, GSSG: oxidized glutathione, MTA: S-methyl-5′-thioadenosine.

	Untreated	Luciferase	Chk-α	PD-L1	Chk-α + PD-L1
C**H**=C**H**	0.90 ± 0.06	0.9 ± 0.1	0.97 ± 0.05	1.28 ± 0.04	1.20 ± 0.07
CH=CH-C**H_2**-	2.7 ± 0.2	2.6 ± 0.3	3.0 ± 0.2	3.80 ± 0.08	3.6 ± 0.2
(CH=CH-C**H_2**-CH=CH)$_n$, n>1	0.61 ± 0.04	0.55 ± 0.07	0.51 ± 0.04	0.81 ± 0.02	0.70 ± 0.06
Linoleic acid	0.61 ± 0.03	0.55 ± 0.07	0.48 ± 0.03	0.81 ± 0.02	0.74 ± 0.05
Glycerol	0.56 ± 0.04	0.51 ± 0.06	0.58 ± 0.03	0.81 ± 0.02	0.67 ± 0.05
PtdEA	0.32 ± 0.03	0.29 ± 0.04	0.24 ± 0.01	0.42 ± 0.03	0.32 ± 0.04
Sphingomyelin	0.13 ± 0.02	0.13 ± 0.02	0.11 ± 0.01	0.21 ± 0.01	0.22 ± 0.02
PtdCho	3.5 ± 0.3	3.3 ± 0.4	3.1 ± 0.4	4.49 ± 0.07	4.1 ± 0.4
Docosahexaenoic acid	0.19 ± 0.01	0.16 ± 0.03	0.12 ± 0.01	0.25 ± 0.01	0.21 ± 0.02
ARA+EPA	0.18 ± 0.03	0.24 ± 0.04	0.020 ± 0.004	0.22 ± 0.05	0.14 ± 0.06
Cholesterol	0.82 ± 0.07	0.56 ± 0.06	0.54 ± 0.09	0.77 ± 0.05	0.65 ± 0.04
CO-C**H_2**-	3.6 ± 0.3	3.6 ± 0.5	3.5 ± 0.2	5.0 ± 0.2	4.4 ± 0.4
CO-CH_2-C**H_2**-	6 ± 1	8 ± 1	3.8 ± 0.2	8 ± 1	8.2 ± 0.7
Lipids (-C**H_2**-)	30 ± 3	33 ± 5	27 ± 1	36 ± 2	36 ± 4
Lipids (-C**H_3**)	21 ± 4	29 ± 4	8.1 ± 0.5	28 ± 5	28 ± 3

Supplementary Table 2. Mean values of lipid metabolites in MDA-MB-231 cells, related to Figure 4.

Values were generated from the quantitative analysis of high-resolution ^{1}H MR spectra obtained at 48h from the lipid phase of MDA-MB-231 that were: untreated (control), transfected with 100 nM luciferase siRNA (Luciferase), transfected with 100 nM Chk-α siRNA (Chk-α), transfected with 100 nM PD-L1 #1 siRNA (PD-L1) and transfected with a mixture of 50 nM PD-L1 and 50 nM Chk-α siRNA(Chk-α + PD-L1). Values represent Mean (a.u.) ± SEM obtained from 3-6 independent experiments.

Lipids (-C**H_3**): methyl groups of fatty acids, Lipids (-C**H_2**-): methylene groups of fatty acids, OOC-C**H_2**: methylene groups at the α position of the carboxylic function, OOC-CH_2-C**H_2**: methylene groups at the β position of the carboxylic function, ARA: arachidonic acid, EPA: eicosapentaenoic acid, PtdEA: phosphatidylethanolamine, PtdCholine: phosphatidylcholine, (CH=CH-C**H_2**-CH=CH)$_n$: diallylic methylene protons, CH=CH-C**H_2**: methylene groups at the α position of a double bond, C**H**=C**H**: fatty acid double bonds.

Tumor type	Chk-α	PD-L1	# samples
Adrenocortical Cancer	10.75 ± 0.08	5.5 ± 0.2	77
Bladder Urothelial Carcinoma	9.89 ± 0.05	7.4 ± 0.1	407
Breast Invasive Carcinoma	10.17 ± 0.02	6.98 ± 0.04	1092
Cervical & Endocervical Cancer	9.26 ± 0.06	8.4 ± 0.1	304
Cholangiocarcinoma	10.5 ± 0.1	6.6 ± 0.2	36
Colon Adenocarcinoma	9.90 ± 0.04	6.62 ± 0.08	288
Diffuse Large B-Cell Lymphoma	9.8 ± 0.1	9.3 ± 0.3	47
Esophageal Carcinoma	10.1 ± 0.1	8.0 ± 0.1	181
Brain Lower Grade Glioma	10.73 ± 0.02	6.24 ± 0.05	509
Glioblastoma Multiforme	10.28 ± 0.05	7.3 ± 0.1	153
Head & Neck Squamous Cell Carcinoma	8.43 ± 0.04	8.55 ± 0.07	518
Kidney Chromophobe	10.77 ± 0.09	8.5 ± 0.2	66
Kidney Clear Cell Carcinoma	9.07 ± 0.03	7.83 ± 0.04	530
Kidney Papillary Cell Carcinoma	9.84 ± 0.04	7.52 ± 0.08	288
Liver Hepatocellular Carcinoma	10.88 ± 0.05	5.94 ± 0.07	369
Lung Adenocarcinoma	10.25 ± 0.04	8.45 ± 0.06	513
Lung Squamous Cell Carcinoma	9.32 ± 0.04	8.78 ± 0.07	498
Mesothelioma	9.18 ± 0.06	7.4 ± 0.2	87
Ovarian Serous Cystadenocarcinoma	10.07 ± 0.04	6.60 ± 0.06	418
Pancreatic Adenocarcinoma	10.51 ± 0.05	7.22 ± 0.08	178
Pheochromocytoma & Paraganglioma	9.42 ± 0.05	7.92 ± 0.09	177
Prostate Adenocarcinoma	11.04 ± 0.03	5.87 ± 0.04	495
Rectum Adenocarcinoma	9.94 ± 0.07	6.5 ± 0.1	92
Sarcoma	9.32 ± 0.05	6.7 ± 0.1	258
Skin Cutaneous Melanoma	10.58 ± 0.07	6.8 ± 0.2	472
Stomach Adenocarcinoma	10.40 ± 0.05	7.78 ± 0.07	414
Testicular Germ Cell Tumor	9.69 ± 0.05	7.5 ± 0.1	148
Thymoma	9.47 ± 0.04	10.0 ± 0.1	119
Thyroid Carcinoma	10.51 ± 0.03	8.12 ± 0.05	504
Uterine Carcinosarcoma	10.35 ± 0.08	5.0 ± 0.2	57
Uterine Corpus Endometrioid Carcinoma	10.62 ± 0.05	6.1 ± 0.1	180
Uveal Melanoma	10.70 ± 0.06	6.4 ± 0.1	79

Supplementary Table 3. Mean values for Chk-α and PD-L1 expression for 32 different tumor types, related to Figure 7.

Values were extracted from the TCGA public database and expressed as the Mean ± SEM. The number of tumor samples for each tumor type available in the TCGA data base are also presented.

Tumor type	Spearman's Correlation	p-Value	q-Value	# samples
Adrenocortical Cancer	-0.26	2.0E-02	8.4E-02	77
Bladder Urothelial Carcinoma	**-0.23**	**2.6E-06**	**8.1E-06**	**407**
Breast Invasive Carcinoma	**-0.13**	**2.3E-05**	**5.1E-05**	**1092**
Cervical & Endocervical Cancer	**-0.45**	**2.8E-16**	**1.8E-14**	**304**
Cholangiocarcinoma	**-0.48**	**3.2E-03**	**6.4E-02**	**36**
Colorn Adenocarcinoma	**-0.26**	**4.0E-07**	**1.7E-06**	**288**
Diffuse Large B-Cell Lymphoma	**-0.51**	**2.0E-04**	**1.5E-03**	**47**
Esophageal Carcinoma	**-0.31**	**1.5E-05**	**3.3E-04**	**181**
Brain Lower Grade Glioma	**-0.37**	**1.1E-18**	**9.5E-18**	**509**
Glioblastoma Multiforme	-0.18	1.7E-02	5.0E-02	153
Head & Neck Squamous Cell Carcinoma	**-0.27**	**8.0E-10**	**8.3E-09**	**5218**
Kidney Chromophobe	**-0.47**	**6.4E-05**	**2.0E-03**	**66**
Kidney Clear Cell Carcinoma	-0.06	1.5E-01	2.2E-01	530
Kidney Papillary Cell Carcinoma	**-0.40**	**7.7E-13**	**1.1E-11**	**288**
Liver Hepatocellular Carcinoma	**-0.27**	**1.4E-07**	**8.9E-07**	**369**
Lung Adenocarcinoma	**-0.39**	**1.9E-20**	**4.5E-19**	**513**
Lung Squamous Cell Carcinoma	**-0.31**	**1.2E-12**	**4.7E-11**	**498**
Mesothelioma	-0.20	6.8E-02	1.9E-01	87
Ovarian Serous Cystadenocarcinoma	-0.04	4.4E-01	6.2E-01	418
Pancreatic Adenocarcinoma	**-0.31**	**2.9E-05**	**1.2E-04**	**178**
Prostate Adenocarcinoma	**-0.32**	**3.9E-13**	**1.4E-12**	**495**
Pheochromocytoma & Paraganglioma	0.14	2.1E-01	3.3E-01	177
Sarcoma	-0.09	1.6E-01	2.7E-01	258
Skin Cutaneous Melanoma	**-0.23**	**7.5E-07**	**4.2E-06**	**472**
Stomach Adenocarcinoma	**-0.28**	**5.8E-09**	**8.0E-08**	**414**
Testicular Germ Cell Tumor	**-0.41**	**1.0E-07**	**9.1E-07**	**148**
Thymoma	**-0.28**	**2.1E-03**	**5.3E-03**	**119**
Thyroid Carcinoma	**-0.62**	**3.8E-56**	**7.2E-54**	**504**
Uterine Carcinosarcoma	-0.17	2.0E-01	5.3E-01	57
Uterine Corpus Endometrioid Carcinoma	-0.21	4.4E-03	2.6E-02	180
Uveal Melanoma	0.00	9.9E-01	9.9E-01	79

Supplementary Table 4. Correlation coefficients between PD-L1 and Chk-α expression in different tumor types, related to Figure 7. Correlation coefficients were calculated used the c-bioportal, and by selecting mRNA Expression Z-scores (RNA Seq V2 RSEM) with a z-score threshold of ±2.0. Statistically significant correlations ($p<0.01$) are highlighted in bold.
